# Superhydrophobic Anticorrosive Phosphonate–Siloxane Films Formed on Zinc with Different Surface Morphology

**DOI:** 10.3390/ma15155360

**Published:** 2022-08-04

**Authors:** Galina V. Redkina, Alexandra S. Sergienko, Yurii I. Kuznetsov, Oleg Yu. Grafov

**Affiliations:** Frumkin Institute of Physical Chemistry and Electrochemistry, Russian Academy of Sciences, Leninsky Prospect 31-4, 119071 Moscow, Russia

**Keywords:** zinc, atmospheric corrosion, passive films, laser processing, SEM, XPS

## Abstract

The composition, structure, and protective and hydrophobic properties of nanoscale films formed layer-by-layer in solutions of sodium dodecylphosphonate (SDDP) and vinyltrimethoxysilane or *n*-octyltriethoxysilane (OTES) on the zinc surface with different morphologies were studied by SEM, XPS, water contact angle measurements, and electrochemical and corrosion tests. The protective, hydrophobic properties of phosphonate–siloxane films on zinc and their stability in a corrosive media are determined both by the initial surface morphology and composition of the surface oxide layer, and by the nature of inhibitors. It was shown that preliminary laser texturing of the zinc surface is preferable than chemical etching to enhance the anticorrosive properties of the resulting thin films. The most stable films with excellent superhydrophobic and protective properties in atmospheres of high humidity and salt spray are formed on the zinc surface with fractal morphology during layer-by-layer passivation with SDDP and OTES.

## 1. Introduction

The protection of metals from corrosion by thin films with a thickness not exceeding several tens of nanometers formed with organic compounds is a current direction in corrosion science and practice [1]. Modern trends in this area are aimed at finding ways to directly design such films, in order to impart multifunctional properties to metal surfaces, namely anticorrosive, antifouling, self-cleaning, and adhesive properties. Along with this, an important requirement for the developed methods of anticorrosion protection and the substances used for this is their environmental and technological safety. This can be achieved using non-toxic organic corrosion inhibitors (OCIs) (Appendix A: Table A1), whose molecules form self-assembled monolayers (SAMs) on the metal surfaces. These OCIs include higher phosphonic and carboxylic acids, thiols, and trialkoxysilanes. All of them are capable of self-assembly on the surface of metals and their oxides, giving them not only anticorrosive, but also hydrophobic properties [2,3,4,5,6,7]. Alkyl phosphonic acids with general formula CH_3_(CH_2_)_n_PO_3_H_2_ (C_n_PAs) are especially promising among them. Their SAMs are more firmly bound to the oxidized metal surface and have greater ordering, density, and stability [2,8,9,10]. These advantages of C_n_PAs are due to the presence of the reactive phosphonic group –PO(OH)_2_ and a hydrophobic alkyl in their molecules. The anchoring group promotes chemisorption of C_n_PAs with the formation of a strong co-ordination bond, while long hydrocarbon radicals are self-assembling, thereby providing hydrophobic properties of the surface and a reliable barrier to the penetration of moisture and corrosive ions.

The specific features of the formation of SAMs in the presence of C_n_PAs and their passivating ability have been most studied in relation to various steels, aluminum, and titanium alloys [2,8,10,11,12,13,14]. There are much fewer studies of their corrosion inhibition of zinc or zinc coatings, and the available studies mainly concern the modification of the zinc oxide surface [9,15,16,17,18,19]. At the same time, such treatment of the zinc surface can increase its corrosion resistance and successfully compete with toxic chromate passivation, which does not meet modern environmental standards [20,21].

We have previously investigated some features of zinc passivation by sodium decyl- and dodecylphosphonic acids (SDDP) to improve its anticorrosive resistance in a chloride-containing aqueous solution and in a humid atmosphere [22,23]. It was shown that alkyl phosphonates chemisorb on the zinc surface from an aqueous solution, forming highly ordered nanoscale layers consisting of ZnO and Zn(OH)_2_ and a phosphonate complex with Zn(II). The stability and protective properties of alkyl phosphonate films depend on several factors: the presence and composition of an oxide–hydroxide layer on the zinc surface, the concentration of the OCIs in the passivating solution, the alkyl length in a C_n_PA molecule, the temperature, and the duration of treatment. We were able to improve the corrosion resistance of zinc by optimizing the treatment conditions, but the resulting phosphonate films are not effective enough for long-term corrosion protection or under more corrosive salt spray conditions.

One of the methods of directed construction of thin films on the metal surfaces to enhance their protective properties can be the combined use of various OCIs by layer-by-layer deposition method. For these purposes, trialkoxysilanes (TAS) with general formula R-(CH_2_)_n_-Si-(OR’)_3_, (where R is an organofunctional radical and R’ is a readily hydrolyzable alkoxy group) are suitable companions for C_n_PAs. Along with low toxicity, they are also capable of self-assembly on the surfaces of many metals, including zinc, imparting upon them valuable properties (hydrophobic, adhesive, anticorrosive, etc.) [24,25,26]. Siloxane films can inhibit the dissolution of zinc in chloride- and sulfate-containing media and under atmospheric conditions, but do not provide complete corrosion suppression [7,27].

The use of TAS to enhance the protective properties of SAMs formed on zinc by other OCIs was shown by K. Aramaki [28]. He used sodium 16-hydroxy-hexadecanate with octyltriethoxysilane or octadecyltriethoxysilane. The resulting ultra-thin films significantly inhibited the cathodic and anodic reactions on zinc in 0.5 M NaCl solution. Studies of the protective properties of sol-gel coatings obtained on low-carbon steel, copper, magnesium, and aluminum alloys in the presence of formulations of phosphonic acids and silanes or synthesized silanes with phosphonic groups also indirectly evidence the possibility of increasing the anti-corrosive resistance of zinc with the combined use of phosphonates and silanes [29,30,31,32,33].

An enhancement of the anticorrosive properties of phosphonate films obtained on zinc in aqueous solution of SDDP by TASs of various structures, as well as some regularities in the formation of such thin phosphonate–siloxane films, were established by us in [34]. The advantage of layer-by-layer treatment with these OCIs over passivation in the aqueous solution of their mixture was shown, as well as the effect of the nature of TAS on the sequence of films formation and their protective properties. In addition, such a sequential treatment of air-oxidized zinc in SDDP and TAS solutions hydrophobizes its surface, but the contact angle (Θ_c_) characterizing the surface wettability with water does not exceed 106 ± 2°.

It is known that the hydrophobic properties of films on the metal surfaces play an important role in their protection against corrosion [35,36]. In order to obtain a superhydrophobic film with Θ_c_ ≥ 150°, the preparation of the metal surface is required, giving it a microstructural (polymodal) roughness. There are many methods for creating such morphology of metal surfaces, including zinc surfaces (electrodeposition, plasma and chemical etching, laser treatment) [37,38,39,40,41]. Among them, nanostructuring of metal surfaces by laser treatment has recently been especially attractive. This method is based on the physical processes of the formation of complex two- and three-dimensional structures with micro- and nanometer sizes on the metal surface under the expose of laser pulses of various intensities and durations on it [42]. Varying the treatment modes allows changing the structure and properties of the surface layer of a metal in a wide range. Such surface treatment of, for example, aluminum and magnesium alloys, followed by heating or adsorption of organic substances improves their anticorrosive resistance in chloride solutions [43,44,45]. However, we did not find any publications on similar studies of zinc or zinc coatings; therefore, we recently showed the possibility of obtaining nanoscale superhydrophobic films on zinc and galvanized steel using laser treatment followed by heating and layer-by-layer passivation with SDDP and vinyltrimethoxysilane or *n*-octyltriethoxysilane [46,47]. The resulting polymodal surface roughness of zinc or galvanized steel made it possible to obtain stable phosphonate–siloxane films with high protective and hydrophobic properties in atmospheres of 100% humidity and salt spray. The proposed passivation treatment of zinc and galvanized steel not only outperforms toxic chromate passivation in efficiency, but is also fire safe, since it involves the use of aqueous (or containing no more than 10% alcohol) solutions of OCIs. At the same time, the features of the influence of different morphology of the zinc surface layer on the properties of films formed by alkyl phosphonate and TAS remained unclear.

In this regard, the purpose of this work was to study and comparatively evaluate the composition, structure, and protective and hydrophobic properties of thin films formed during layer-by-layer passivation of SDDP and TASs on the zinc surface with different morphology, which was obtained by laser treatment and simple chemical etching.

## 2. Materials and Methods

### 2.1. Materials

The studies were carried out on zinc samples containing 99.975% Zn and impurities (Fe, Al, Cu, Sn, Pb, Cd, As) up to 0.025%. Phosphonate–siloxane films on zinc were obtained by layer-by-layer passivation in solutions of sodium salt of dodecylphosphonic acid CH_3_-(CH_2_)_11_-PO(ONa)_2_ (SDDP) and TAS, namely vinyltrimethoxysilane H_2_C=CH-Si(OCH_3_)_3_ (VTMS) and *n*-octyltriethoxysilane CH_3_-(CH_2_)_7_-Si(OC_2_H_5_)_3_ (OTES). SDDP was used in the form of an aqueous solution prepared by dissolving powder of dodecylphosphonic acid (98.0%, TCI, Tokyo, Japan) in distilled water and neutralizing it with NaOH when heated to 60 °C. TASs (97.0%, Sigma Aldrich, Darmstadt, Germany) was used in the form of water–alcohol solutions with ethanol content of 10%.

### 2.2. Preparation of Anticorrosion Superhydrophobic Films on the Zinc Surface

The composition, structure, and protective and hydrophobic properties of phosphonate–siloxane films formed on four different types of zinc surface were studied in this work:

- “Smooth” surface, oxidized in air (Type 1);
- Textured surface by chemical etching (Type 2);
- Textured surface by laser (Types 3 and 4).

In the first case (Type 1), zinc samples with a size of 25 × 15 × 6 mm were polished with 240–2500 grit silicon carbide abrasive paper (Metkon, Bursa, Turkey), degreased with acetone, and kept in air for 1 h to form an oxide layer to ensure a reproducible surface condition. To obtain the Type 2 surface, the samples were etched in 4.0 M HCl at room temperature for 3 min with stirring of the solution. After that, the samples were washed in distilled water assisted with ultrasound and dried in an oven at 150 °C for 1 h. A similar method for texturing the zinc surface is described in [40].

Laser texturing of the surface of zinc samples was carried out using ytterbium short-pulsed fiber laser XM-30 (Kazan, Russia) with the following parameters: λ = 1.064 µm; *p* = 7.5 W; υ = 20 kHz; d = 0.01 mm; l = 0.01 mm, where λ is the wavelength, *p* is the radiation power, υ is the radiation frequency, d is the laser beam diameter, and l is the distance between two adjacent linear trajectories. Laser scanning was performed on a grid. In order to form nano/microstructures of various sizes and structures on the zinc surface, the laser scanning speed (V) was varied: 100 (Type 4) and 700 mm/s (Type 3). The zinc surface became black “velvet” in the first case and matt light gray in the second case. After laser treatment, the samples were cleaned in acetone assisted with ultrasound for 60 s to remove particles formed that were weakly bound to the metal surface. Then, the samples were kept in an oven at 150 °C for 1 h.

Prepared zinc samples with different surface morphologies were treated in 2.5 mM SDDP solution and/or 10.0 mM TAS solutions at 40 °C and constant stirring speed of 250 rpm. The samples were dried in an oven at 150 °C for 1 h after treatment in TAS solutions. In the joint treatment of zinc with SDDP and TAS, the layer-by-layer method of thin films formation was used. The duration of exposure in each solution was 1 h with intermediate drying at 60 °C for 30 min. We previously determined these conditions of the zinc passivation with SDDP and TAS as optimal in terms of the efficiency of anticorrosion protection [34].

### 2.3. Microstructural and Chemical Analysis

The surface morphology of Zn samples was studied using a TESCAN VEGA LMH scanning electron microscope with a LaB6 cathode (SEM) and an Oxford Instruments Advanced AZtecEnergy energy dispersive X-ray (EDX) microanalysis system, including INCA Energy 350/X-max 50 with X-Max 50 Standard nitrogen-free detector (silicon drift detecting element with an active area of 50 mm^2^; resolution at the MnKα line is 127 eV). The penetration depth of electrons into the sample is about 0.2 μm at a voltage of 20 kV. The standard error of measurement the percentage of elements in the samples did not exceed 0.1 wt%. Pure zinc with a Zn percentage of 99.9999 wt% was used as a reference. The qualitative elemental composition of microvolumes was determined by comparing the characteristic radiation wavelengths observed and stored in the analyzer. Quantitative analysis was carried out on the basis of differences in the radiation intensity of the strongest line of the K- or L-series of the characteristic radiation of the element being determined.

The surface roughness of the zinc samples was measured by the probe method using a Model-130 profilometer (Proton, Zelenograd, Russia). As a result, the values of the roughness parameters were obtained: *R*_z_ is the height of profile irregularities at ten points (the sum of the average absolute height values of the five largest profile projections, and the depths of the five deepest depressions of the profile within the base length), μm; *R*_a_ is the mean arithmetic deviation of the profile, μm; *S* is the average step of the local projections of the profile within the base length, μm. The roughness class was determined according to ISO 1302:2002 based on the obtained parameters [48].

Qualitative and quantitative analysis of phosphonate–siloxane films obtained on “smooth” and textured zinc surfaces was performed by X-ray photoelectron spectroscopy (XPS). Zinc samples were prepared as described in Section 2.2 and then washed in distilled water with ultrasound to remove physically adsorbed molecules. XPS studies were carried out using an Omicron ESCA + X-ray photoelectron spectrometer (OMICRON, Germany). The pressure in the analyzer chamber did not exceed 8 × 10^−9^ mbar; an aluminum anode (AlKα 1486.6 eV, 252 W) served as the radiation source. The pass energy of the analyzer was 20 eV. A neutralizer was used to prevent samples from charging. The positions of the spectra were standardized with respect to the C1*s* peak of hydrocarbon impurities from the atmosphere with a maximum at 285.0 eV. The spectra were decomposed into components after subtracting the background determined by the Shirley method [49]. The XPS MultiQuant program was used to determine the thickness of the layers formed on the zinc surface [50]. In the quantitative analysis, we used the Scofield sensitivity coefficients [51], which were corrected by the analysis of salts with a known composition, as well as the mean free paths of electrons located on these shells.

### 2.4. Study of the Protective and Hydrophobic Properties of Films

The resistance of phosphonate–siloxane films on zinc to the corrosive action of chlorides was studied by the potentiodynamic polarization method using an Autolab PGSTAT302N potentiostat/galvanostat (Autolab, Utrecht, The Netherlands) with the potential scan rate of 0.002 V/s. The measurements were performed in a borate buffer solution with pH 7.38 ± 2 (0.2 M H_3_BO_3_ + 0.05 M Na_2_B_2_O_7_ × 10 H_2_O) containing 0.001 and 0.5 M NaCl (chemically pure, RusKhim, Moscow, Russia). The reference electrode was a two-key saturated silver chloride electrode, and a counter electrode was a pyrographite plate. The measured electrode potentials (*E*) were converted to the normal hydrogen scale. The working area of the electrodes was 1.0 cm^2^. All electrochemical measurements were performed under static conditions at room temperature of the solution (23 ± 2 °C) and with natural aeration. The efficiency of zinc protection against corrosion was judged by the value of the difference of local depassivation potentials ($\Delta E_{\mathrm{pit}}=E_{\mathrm{pit}}^{\mathrm{OCI}}-E_{\mathrm{pit}}^{0}$), which were determined from the anodic polarization curves on samples without treatment ($E_{\mathrm{pit}}^{0}$) and with preliminary passivation with OCIs ($E_{\mathrm{pit}}^{\mathrm{OCI}}$).

The protective properties of phosphonate–siloxane films on zinc in corrosive atmospheres were evaluated by the results of direct corrosion tests in a heat and moisture chamber (KTV-0.1-002, Volgograd, Russia) and in a salt spray chamber (Weiss SC/KWT 450, Reiskirchen, Germany) according to ISO 4536:1985 and ISO 9227:2017 [52,53]. In the first case, the tests were carried out under severe conditions of 100% humidity with daily condensation of moisture on the samples; in the second case, in an atmosphere of neutral salt spray, where a solution of 5.0% NaCl (pH 6.5–7.2) was sprayed. Visual inspection of the samples was carried out at regular intervals to determine the time of appearance of the first corrosion damage (*τ*_cor_). The criteria for the efficiency of the anticorrosion protection of zinc by the obtained films were the coefficient of corrosion retardation (**γ**) and the degree of protection (*Z*), which were calculated using the following formulas:

(1) $$\gamma =\frac{\tau_{\mathrm{cor}}^{\mathrm{pas}}}{\tau_{\mathrm{cor}}^{0}}$$

where $\tau_{\mathrm{cor}}^{0}$—time of appearance of the first corrosion damage on the zinc surface without passivation and $\tau_{\mathrm{cor}}^{\mathrm{pas}}$—time of appearance of the first corrosion damage on the zinc surface after passivation with OCIs;

(2) $$Z\%=\left(1-\frac{1}{\gamma }\right)\cdot 100$$

The hydrophobic properties of the films and their stability were judged by the value of the static contact angle (Θ_c_) of a droplet of distilled water and its change in time during corrosion tests of zinc samples in a heat and moisture chamber. The Θ_c_ values were calculated in a graphical editor using photographic images of a drop obtained using a laboratory unit with a microscope and integrated M1000 PLUS camera (Levenhuk, Tampa, FL, USA). The droplet volume was 3–5 μL in each measurement. The standard deviation of Θ_c_ was 1.0–2.0°.

## 3. Results and Discussion

### 3.1. Morphology of Zinc Surface

It is known that the morphology of the metal surface has a significant effect on its corrosion behavior. With an increase in the micro-irregularities height, the corrosion of the metal, including its tendency to local destruction, intensifies. Along with this, the microstructural roughness of the metal surface is a necessary condition for its superhydrophobization with OCIs. Variation in the morphology and surface roughness parameters can obviously affect the hydrophobic and anticorrosion properties of thin films formed in the presence of OICs. In order to understand the relationship between surface morphology and protective properties of the obtained superhydrophobic films, we analyzed the roughness parameters and SEM images of four types of zinc surface.

**Figure 1:**
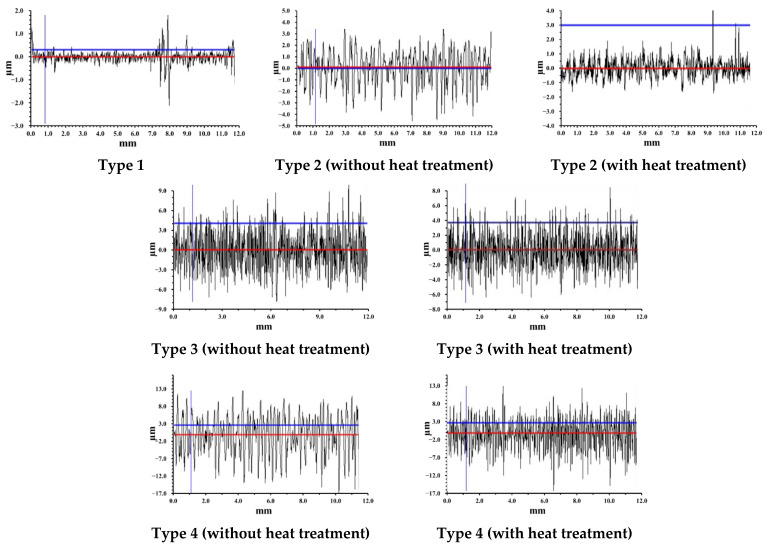
Profilograms of different types of zinc surface (the red line is the profile base line, blue lines are measuring lines).

The results of measuring the roughness parameters of zinc surface types studied in this work by the profilometric method are presented in Table 1 and Figure 1. The “smooth” air-oxidized zinc surface (Type 1) corresponds to the third roughness class [48].

Etching and laser treatment lead to an increase in its inhomogeneity. The R_z_ value and the roughness class of various zinc surfaces increase in the following order: Type 1 < Type 2 < Type 3 < Type 4, and reach the highest value when laser treatment at low laser scanning speed. In this case, the S value upon laser texturing of the zinc surface increases insignificantly, by 1.4–1.8 times. On the contrary, after zinc etching, along with an increase in roughness, the S value increases significantly up to 21.32 μm. The obtained roughness parameters for various textured zinc surfaces are consistent with the results of the analysis of their morphology by the SEM method.

**Figure 2:**
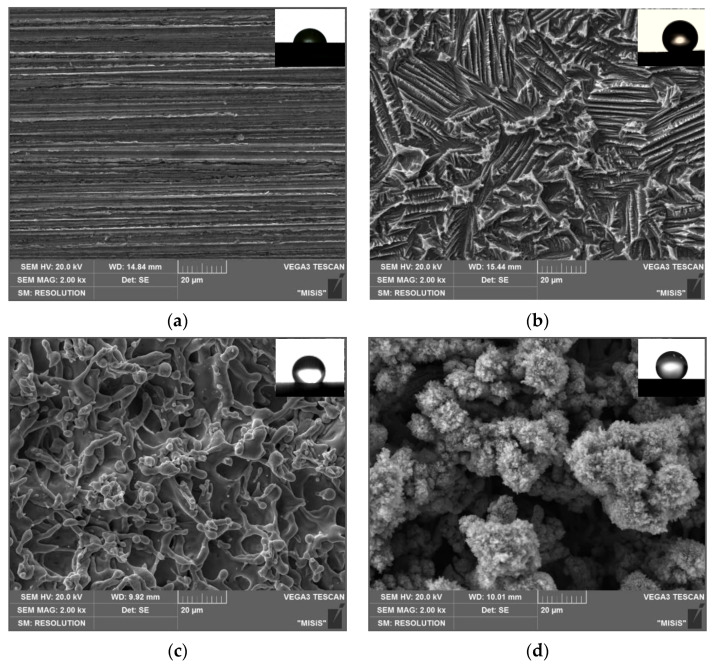
SEM images of (**a**) “smooth” zinc surface (Type 1) and after texturing by (**b**) chemical etching (Type 2) and laser with (**c**) *V* = 700 mm/s (Type 3) and (**d**) 100 mm/s (Type 4).

Analysis of the microstructure of the zinc surface of Type 1 (Figure 2a) shows that there are no damages and defects on the sample. Microscale hillocks and grooves are observed on the zinc surface etched in HCl solution (Type 2), which form a special hierarchical structure (Figure 2b). The hillocks are 10–30 µm in width and are dispersed over the zinc surface, which correlates well with the results of profilometry. It can be seen that the dispersed hillocks are formed by etching the grains along the boundaries. The microgrooves are parallel to each other, pass through each slope of the hillocks and are distributed along the boundaries of the former zinc grains.

The microstructure of the zinc surface of Types 3 and 4 (Figure 2c,d) obtained by laser treatment is fundamentally different from the etched textured surface (Type 2). The interaction of the laser beam with the zinc surface is accompanied by local heating followed by rapid cooling of the surface layer, which leads to laser ablation, metal oxidation, grain dispersion, and accumulation of surface stresses. In addition, particles formed in plasma as a result of laser ablation of surface layers are partially deposited on the treated surface. At high laser scanning speed (Type 3) (Figure 2c), the surface structures are flatter and smaller. A decrease in the V value from 700 to 100 mm/s makes it possible to maintain the molten state of the surface layers much longer in the presence of oxygen (Type 4, Figure 2d). This leads to the formation of a thicker oxide layer on the zinc surface. Upon repeated passage of the laser beam, the oxide film is destroyed during melting and moves to the inner layers due to convection, while the oxidation of the freshly exposed top layer begins again. The metal goes through several stages of melting and solidification, which leads to excessive oxidation and the formation of a thick surface oxide layer covered with a layer of nanoparticles ejected from ablation craters. As a result, a fractal surface is formed, represented by coral-like surface structures. The morphology characteristic of the zinc surface of Type 4 demonstrates the features of polymodal roughness. It should be noted that such laser treatment gives the zinc surface a black color. According to the results of EDX analysis, the blackening of the surface is not associated with a change in the elemental composition (Table 2). The authors came to similar conclusions in [54,55]. They obtained black “velvet” multifunctional surfaces of brass, platinum, titanium, and its alloy with nickel by creating a hierarchical nano/microstructure by femtosecond laser pulses. Such surfaces had light-absorbing, superhydrophobic, and self-cleaning properties. The coral-like surface structures significantly increase the metal surface area, and a high degree of roughness makes them a porous light absorber owing to which the surface appearance acquires a coal-black color.

After texturing the zinc surface by any of the above methods, we carried out its heat treatment. This stage of preparation of metal surfaces during their superhydrophobization, described in [43,44] for copper and magnesium alloy, was tested by us earlier for the zinc surface in [46]. The effect of heat treatment on the morphology and composition of the surface layer of zinc was evaluated by the results of EDX analysis surfaces of Types 3 and 4. It can be seen from Table 2 that after laser exposure followed by heat treatment, the oxygen content decreases by 0.65–0.98 wt%. Presumably when the zinc sample is heated to 150 °C, the fraction of Zn(OH)_2_ decreases in the surface layer as a result of the transformation to ZnO, thereby increasing its fraction. As a result, smaller and more compact surface structures are formed, consisting mainly of ZnO particles. This is consistent with profilometric data showing a slight decrease in the roughness parameters on laser textured zinc samples after heat treatment. It can also be seen that the amount of oxygen is approximately 10 times greater on the zinc surface of Type 4 than on all other surface types (Table 2). This is due to the large specific area of such a fractal surface due to the formation of a larger number of smaller nanoparticles, each of which is covered with an oxide layer.

### 3.2. Hydrophobic and Protective Properties of Phosphonate–Siloxane Films on Zinc

**Figure 3:**
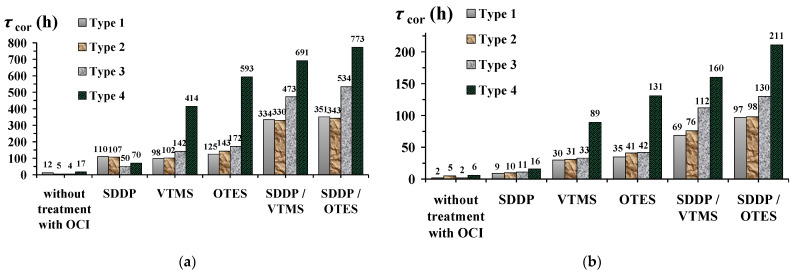
*τ*_cor_ values (**a**) in the heat and moisture chamber and in a salt spray chamber (**b**) on “smooth” (Type 1) and textured (Type 2–4) zinc samples with subsequent treatment in SDDP and TASs solutions. The concentrations of OCI solutions are given in mM.

Table 3 shows the results of measurements of Θ_c_ depending on the type of zinc surface morphology and the composition of the inhibiting solution. The “smooth” air-oxidized zinc surface is hydrophilic and the Θ_c_ value does not exceed 72 ± 3°. Chemical or laser texturing with subsequent heating gives it hydrophobic properties even without treatment with OCIs. The Θ_c_ value varies from 125 to 155° depending on the surface morphology and takes the greatest value for a surface with polymodal morphology (Type 4). Despite the hydrophobic properties, the textured zinc surfaces quickly corrode in heat and moisture or salt spray chambers (Figure 3). The adsorption of SDDP or TAS on such surfaces enhances their hydrophobicity in comparison with the “smooth” surface modified with the OCIs. In the case of treatment of Type 4 surface in OTES solution, the Θ_c_ value even reaches 159°. As with layer-by-layer passivation of the “smooth” surface, a similar treatment of previously textured zinc samples in solutions of SDDP and any of the TASs studied leads to an enhance in the hydrophobic properties of the formed films. However, superhydrophobic films are obtained only by treatment SDDP with OTES. At the same time, the highest Θ_c_ value (165°) is observed on the zinc surface with a larger specific area (Type 4). On the one hand, this is due to the difference in the structure and hydrophobicity of TAS molecules. The calculation of the logarithm of the distribution coefficient of the substance in the system of two immiscible liquids (octanol–water) (log*P*), characterizing the hydrophobicity of compounds [56], showed that it is 3.12 ± 0.66 for VTMS and 5.45 ± 0.42 for OTES. Thus, OTES is more hydrophobic, has a relatively long alkyl in the molecule and is capable of forming the dense ordered self-assembled layer on the metal surface. On the other hand, the superhydrophobic state of the Type 4 surface is due to the polymodal roughness. The heterogeneous wetting regime is realized on such fractal surfaces, in which the filling of voids between micro/nanostructures with water is problematic [57]. In this case, the Cassie–Baxter equation is valid [36]:

cosΘ_c_ = *f*_1_ ∙ cosΘ_c,1_ + *f*_2_ ∙ cosΘ_c,2_ (3)

where *f*_1_ and *f*_2_ are surface area fractions of polymodal structures and the trapped air in the voids between them that are in contact with the liquid, respectively; Θ_c,1_ is the water contact angle on a “smooth” surface; Θ_c,2_ is the water contact angle on the surface of pores with air. Given that *f*_1_ + *f*_1_ = 1, Θ_c,1_ = 72°, Θ_c,2_ = 180°, and Θ_c_ = 165°, *f*_1_ and *f*_2_ are 0.026 and 0.974, respectively. Thus, when a liquid hits a superhydrophobic zinc surface, on average, only 2.6% of the surface is in contact with the liquid drop, and the remaining 97.4% correspond to the liquid droplet contact area with air. The small area of contact between the liquid and the solid surface can improve the corrosion resistance of the zinc surface with a phosphonate–siloxane film in humid atmospheres.

The results of corrosion tests in heat and moisture and salt spray chambers showed that films obtained on laser textured zinc surfaces (Types 3 and 4) during layer-by-layer passivation in solutions of SDDP and TASs significantly superior in protective properties of films formed on etched or “smooth” surfaces (Types 2 and 1) (Figure 3, Table 4). The inhibiting efficiency of the latter was studied by us earlier in [34]. Sequential passivation with SDDP and TAS is more effective than with individual OCIs. At the same time, films obtained with OTES had better protective properties than with VTMS. This is due to the different structure of TASs, which, as is known, greatly affects their inhibiting ability. With an increase in the length of the hydrocarbon radical in a TAS molecule, not only its hydrophobicity, and, consequently, the surface activity, but also the stability of the resulting siloxane films enhance. In addition, not only the higher hydrophobicity of the films formed in the presence of SDDP and OTES, but also their greater thickness can explain their better anticorrosion properties. This is evidenced by our earlier results of ex situ ellipsometric measurements on galvanized steel [47]. It has been shown that exactly the same layer-by-layer treatment of galvanized steel in solutions of SDDP and VTMS or OTES leads to the formation of nanoscale protective films with thicknesses of 61.5 and 70.0 ± 2.5 nm, respectively. In this case, the thickness of the first chemisorbed phosphonate layer is 9.4 ± 0.6 nm, which, as will be shown below, is in good agreement with the results of XPS analysis of similar films on zinc. Along with this, superhydrophobic layers impart a negative charge to the surface, hindering the adsorption of chloride ions and oxygen, thereby inhibiting zinc corrosion [26]. Thus, in the presence of SDDP and the more hydrophobic OTES, a negatively charged superhydrophobic film is formed on the zinc surface, which consists of a siloxane matrix that blocks existing defects in the film formed by the chemisorbed alkyl phosphonate.

Among the studied types of zinc surface, the surface with polymodal morphology (Type 4) obtained by laser treatment at a low scanning speed provided the highest anticorrosive properties of the film formed in SDDP and OTES solutions. Such film enhanced the corrosion resistance of zinc in an atmosphere of 100% humidity with daily condensation of moisture on the samples by almost 64.4 times (Figure 3a, Table 4). Even under more corrosive salt spray conditions, the coefficient of corrosion retardation calculated from the *τ*_cor_ value was 105.5 (Figure 3b, Table 4). This corresponds to a high degree of zinc protection under the studied conditions (98.4 and 99.1%, respectively). Note that after the appearance of small corrosion pockets on the zinc surface, it still remained superhydrophobic.

At the same time, hydrophobization, or even superhydrophobization, of zinc with phosphonate–siloxane films does not always ensure its high anticorrosive resistance in corrosive atmospheres. For example, the morphology of the zinc surface obtained by etching (Type 2) makes it possible to obtain films in SDDP and TASs solutions that are not inferior in their hydrophobic properties to those formed on a laser textured surface of Type 3. However, their anticorrosive efficiency is 1.5 ± 0.1 times lower (judging by the γ values) and practically does not differ from the efficiency of films obtained on a “smooth” zinc surface (Type 1) (Table 4). This is due to the fundamentally different morphology of the zinc surface after etching and laser treatment, in particular, the sizes of microstructures. Despite the increase in the roughness of the zinc surface after etching (Type 2), it is characterized by the presence of relatively large microstructures in the form of dispersed grooves and hillocks 10–30 µm in width. Salt spray droplets are smaller in size (1–10 µm in diameter [53]), so they condense in microstructures on the surface and a gas interlayer is not formed. Thus, the anticorrosive resistance of such surfaces is provided only by the properties of a thin phosphonate–siloxane film, as well as on a “smooth” surface (Type 1).

**Figure 4:**
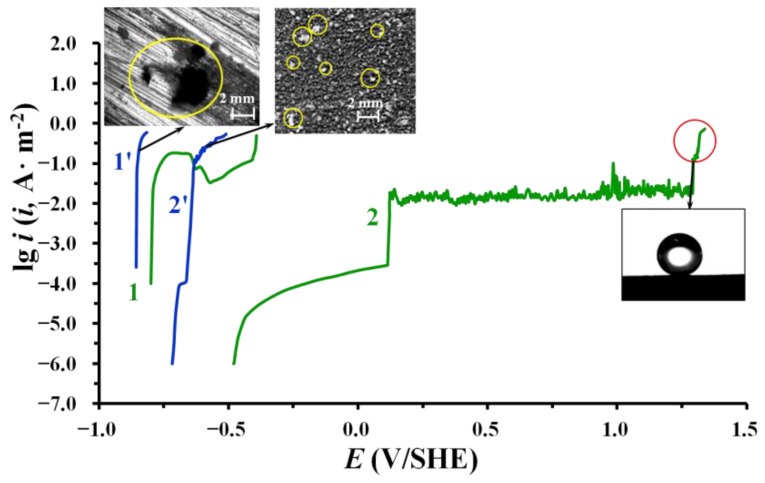
Anodic polarization curves of zinc in borate buffer solution (pH 7.4) containing (1,2) 0.001 and (1′,2′) 0.5 M of NaCl, (1,1′) without any treatment and (2,2′) after laser and layer-by-layer treatment in solutions of 2.5 mM SDDP and 10.0 mM OTES.

Additional information about the protective properties of the obtained superhydrophobic films, in particular their resistance to the corrosive action of Cl^−^ ions, can be obtained from the results of polarization measurements in aqueous solutions with different concentrations of NaCl. Analysis of the anode polarization curves obtained in a borate buffer solution containing 0.001 M NaCl showed that the film formed on the zinc surface of Type 4 by layer-by-layer treatment with SDDP and OTES provide its passivation. This is evidenced by a shift in the corrosion potential (*E*_cor_) to the passive region (from −0.80 V to −0.48 V). Such a film effectively prevents local destruction of zinc compared to an untreated with OCIs “smooth” electrode coated with an air-formed oxide film (∆*E*_pit_ = 0.54 V) (Figure 4, curve 2). In this case, current oscillations are observed on the anodic polarization curve at *E* > 0.11 V. In this region, the anode current density does not depend on *E* and does not exceed 0.02 A/m^2^, which indicates a low dissolution rate of zinc. Despite this, it cannot be called passive, because the processes of formation and repassivation of fine pits take place here, the presence of which we observed on the surface of the zinc electrode when it was removed from the solution. It is more correct to call this region the quasi-passive state region. Local destruction of the protective film can occur in the most vulnerable places, for example, on the protrusions of the metal surface with polymodal morphology. It should be noted that the surface of the zinc electrode still remains superhydrophobic. Apparently, the size of the corrosion damage is so small that they do not violate the integrity of the superhydrophobic film. Such a quasi-passivity region on the anode curve is observed up to *E* = 1.30 V, after which there is a sharp increase in the anode current, indicating a significant destruction of the phosphonate–siloxane protective film on zinc.

With an increase in the NaCl concentration in an aqueous solution to 0.5 M, the protective properties of the phosphonate–siloxane film noticeably decrease, judging by the shift in the *E*_cor_ and *E*_pit_ of zinc to the region of negative values (−0.72 0 V and −0.67 V, respectively) (Figure 4, curve 2′). However, even under these conditions, a significant slowdown of the anode process is observed in comparison with the bare (untreated) zinc surface (Type 1), for which *E*_cor_ = −0.86 V. It is important to note that after electrochemical tests under these conditions, large local damage with a lot of corrosion products were observed on the untreated zinc electrode, while pits were fine but evenly distributed over the electrode surface with a superhydrophobic anticorrosive film. Thus, even in such a corrosive medium close to seawater, the proposed method of zinc treatment inhibits its anodic dissolution. It should be noted that the evaluation of the protective properties of superhydrophobic films by electrochemical tests in an aqueous solution is only an additional method and cannot replace long-term corrosion tests under atmospheric conditions. This is due to the fact that the fractal surfaces, which is the zinc surface of Type 4, are characterized by the presence of a gas interlayer at the solid/liquid interface. During anodic polarization, it performs, in fact, the role of a dielectric layer that prevents the dissolution of the metal.

**Figure 5:**
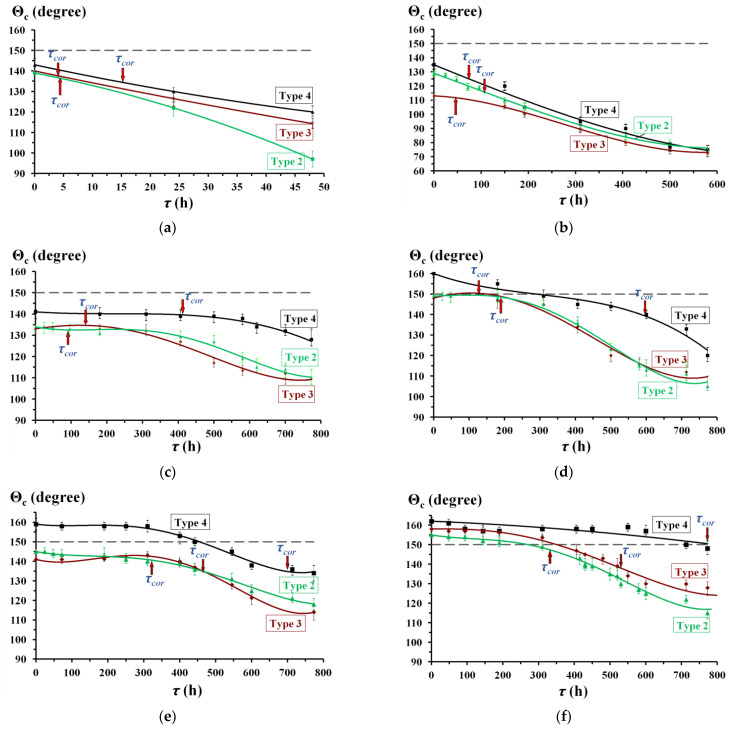
Change in the Θ_c_ value with time (*τ*) on textured surfaces of zinc samples (Type 2–4) after layer-by-layer treatment in solutions of OCIs: (**a**)—without treatment with OCIs; (**b**)—2.5 mM SDDP; (**c**)—10.0 mM VTMS; (**d**)—10.0 mM OTES; (**e**)—2.5 mM SDDP///10.0 mM VTMS; (**f**)—2.5 mM SDDP///10.0 mM OTES.

An important indicator of the efficiency of superhydrophobic films and the possibility of their practical application is the ability to maintain their properties under operating conditions. We carried out an evaluation of film degradation, i.e., of the loss of hydrophobic properties, in parallel with corrosion tests in a chamber of heat and moisture. Figure 5 shows the change of the Θ_c_ value with time for all types of textured zinc surfaces treated both with individual OCIs and successively. A comparative evaluation of these results shows that the layer-by-layer passivation with SDDP and TASs provides more stable hydrophobic properties of protective films regardless of the morphology of the zinc surface. Phosphonate–siloxane films obtained on zinc surfaces textured by etching and laser at a high scanning speed (Types 2 and 3) are characterized by similar nature of degradation in time, despite a significant difference in protective properties. This can be explained by the fact that the size of a water droplet when measured Θ_c_ exceeds the size of liquid droplets condensing on the surface during corrosion tests. In this regard, moisture droplets can condense in microstructures of the Type 2 zinc surface, which are relatively large. In this case, a gas interlayer is not formed and corrosion damages appear much earlier than a significant decrease in the value Θ_c_ is observed. Similar films on the surface with fractal morphology (Type 4) have the most stable superhydrophobic properties. At the same time, the use of OTES together with SDDP is more efficient than VTMS, since even with prolonged contact with a corrosive medium (more than 700 h), the Cassi–Baxter state is preserved (Θ_c_ = 150 ± 4°). The degradation of such film is nonuniform. Even in the presence of small local corrosion damages, the zinc surface remains superhydrophobic.

### 3.3. XPS Studies of the Chemical Composition of Phosphonate-Siloxane Films on Zinc

**Figure 6:**
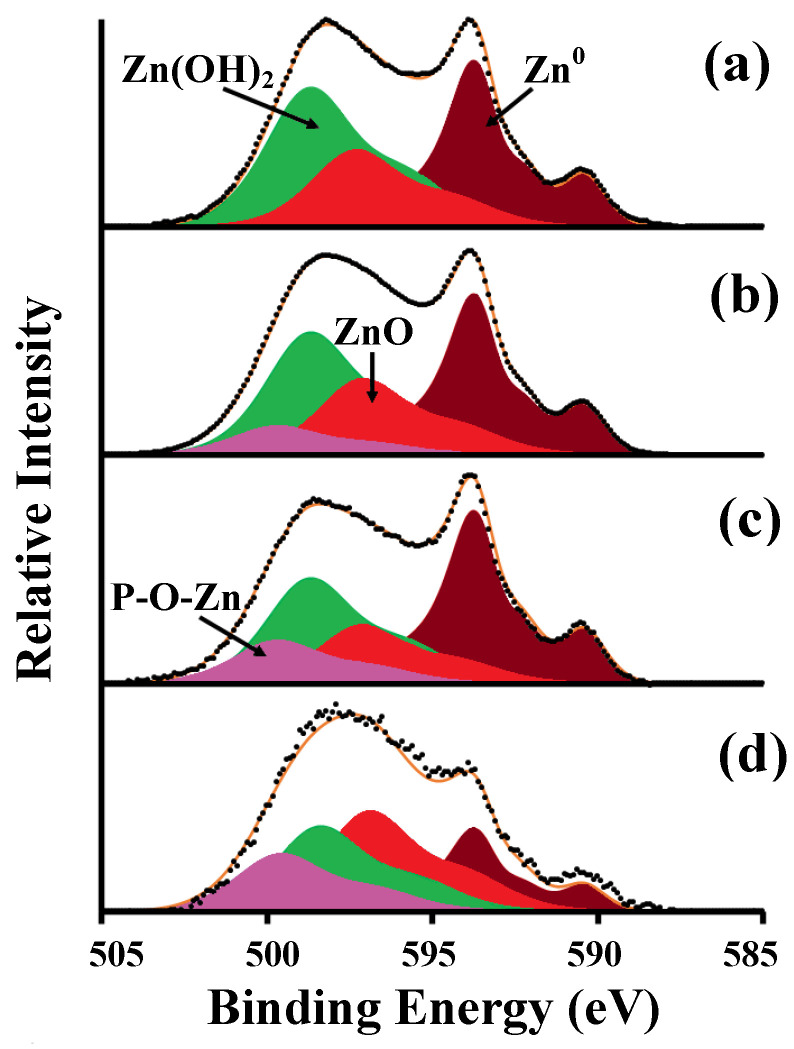
Auger ZnL_3_M_45_M_45_ spectra of zinc samples with “smooth” surface (Type 1) (**a**) before and (**b**–**d**) after treatment in solution of 2.5 mM SDDP. The electron emission angle relative to the sample surface: (**a**,**b**) 90, (**c**) 50, and (**d**) 15°.

The influence of surface morphology on the composition and structure of protective layers was studied using the example of layer-by-layer passivation of zinc in solutions of SDDP and OTES, which showed the highest anticorrosive efficiency. To determine the positions of the peaks of the states of the elements, the obtained spectra were compared with the spectra of zinc samples with protective layers formed with individual OCIs. For qualitative analysis of zinc states, the Auger spectrum of ZnL_3_M_45_M_45_ electrons was used. This is due to the low information content of the Zn2*p*_3/2_ spectrum, since the positions of singlets for metallic zinc Zn^0^ (1021.7 eV) and its oxide ZnO (1022.1 eV) differ by only 0.4 eV, which makes it difficult to identify them when they are both present on the surface [58,59,60]. In the Auger ZnL_3_M_45_M_45_ spectrum (Figure 6a), obtained on the bare zinc surface (Type 1), three states can be clearly distinguished, namely Zn^0^ (493.7 eV); ZnO (497.1 eV); Zn(OH)_2_ (498.5 eV). According to calculations in the XPS MultiQuant program, the thicknesses of the surface layers were 1.8 ± 0.2 and 1.5 ± 0.2 nm for Zn(OH)_2_ and ZnO, respectively.

**Figure 7:**
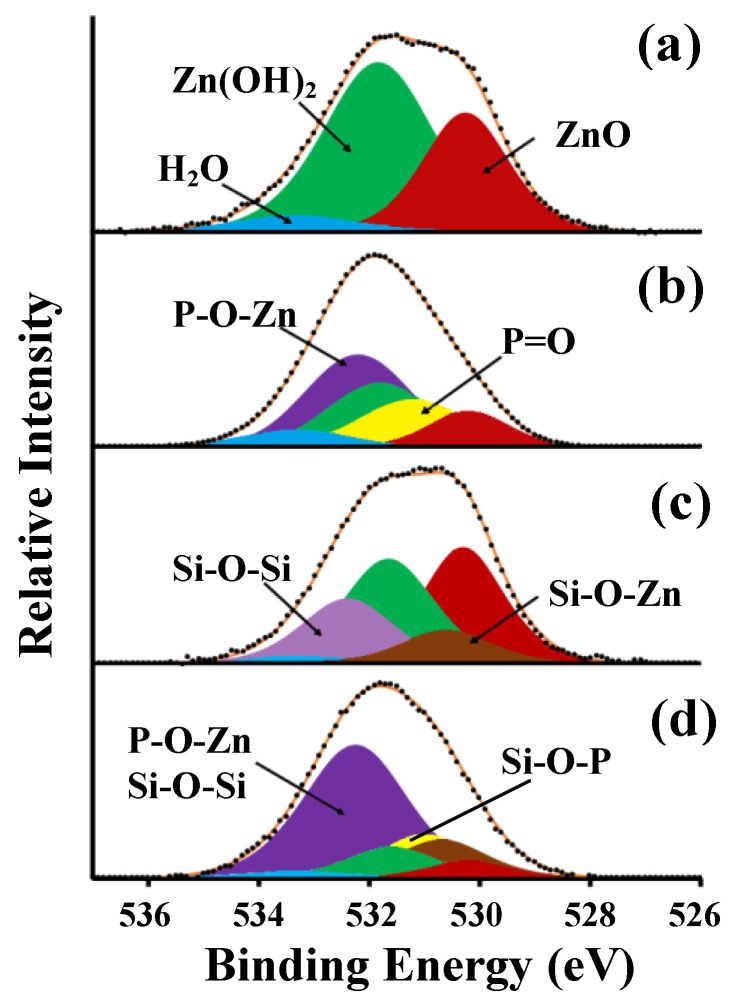
O1*s* spectra of zinc samples with “smooth” surface (Type 1) (**a**) before and after treatment in solutions of (**b**) 2.5 mM SDDP, (**c**) 10.0 mM OTES, (**d**) 2.5 mM SDDP/10.0 mM OTES.

After treatment of zinc in SDDP solution, in the Auger ZnL_3_M_45_M_45_ spectrum there is a contortion in the region of higher binding energies, which can be isolated as a separate component with a maximum at 499.6 eV (Figure 6b). The intensity of this peak increases with decreasing electron departure angle relative to the sample surface (Figure 6b–d). This component may be due to the hardly soluble zinc phosphonate complex (ZnDDP) formed during the interaction of the bidentate phosphonic group of SDDP with the oxidized zinc surface, i.e., the formation of the strong coordination bond P-O-Zn. The chemisorption of SDDP on the zinc surface is also indicated by the appearance of the peak of P2*p* electrons in the spectrum, which does not disappear after ultrasonic washing. The binding energy (*E*_b_) of P2*p* electrons is 133.6 eV, which is slightly lower than in the crystalline SDDP (134.2 eV). An asymmetric peak appears in the spectrum of C1*s* electrons, which can be decomposed into two components: the maximum at *E*_b_ = 285.0 eV corresponds to the C-H bond, and at *E*_b_ = 286.9 eV corresponds to the C-P bond. Two additional peaks appear in the spectrum of O1*s* electrons after treatment in a SDDP solution, the intensities of which are related to each other as 2:1. These peaks correspond to P-O-Zn (532.4 eV) and P=O (531.2 eV) bonds, along with peaks attributed to ZnO (530.2 eV), Zn(OH)_2_ (531.8 eV), and adsorbed H_2_O_ads_ (533.3 eV) (Figure 7a,b).

Using the XPS MultiQuant program and the integrated intensities of the C1*s*, O1*s*, P2*p*, and Zn2*p* peaks, the layer thicknesses on the zinc surface (Type 1) after treatment in a SDDP solution were calculated. The intensity of the Zn2*p* doublet was divided in proportion to the intensities of the corresponding components of the Auger ZnL_3_M_45_M_45_ spectrum. According to the calculations, the layer thicknesses were 5.4 ± 0.2 nm for ZnDDP, 1.8 ± 0.2 and 0.3 ± 0.2 nm for Zn(OH)_2_ and ZnO, respectively. Considering that the thickness of the ZnDDP layer exceeds the length of the SDDP molecule even after ultrasonic washing, it is possible to assume the formation of a strongly chemisorbed polymolecular layer. This layer can be retained due to the formation of a strongly chemisorbed (-Zn-O-P-O-Zn-O-P-) chain complex on the surface.

Note that the observed layer thicknesses are at the limit of the possible measurement depth in the XPS, which may cause low values of the ZnO thickness. Analysis of the XPS spectra obtained at different electron emission angles relative to the sample surface (50° and 90°) showed that the [C]/[P] ratio is 12.31 and 11.47, respectively. Considering that according to stoichiometry in the SDDP molecule the ratio [C]/[P] equal to 12:1, the angle between the alkyl chains in the phosphonate layer and the normal to the surface (tilt angle) is 25°.

The results obtained are consistent with the mechanism of the formation of protective layers on metals by alkyl phosphonates in neutral aqueous media. It is based on the possibility of nucleophilic substitution of a hydroxyl with an organic anion on the metal surface [1,2]. This process in the presence of SDDP on an oxidized zinc surface can be expressed in a simplified form by the following reaction:

mZn(OH)_2_ + n[R-PO_3_]^2−^ → Zn_m_[R-PO_3_]_n_ +2OH^−^ (4)

where R is hydrocarbon radical. As a result, a protective film is formed, which is firmly bonded to the metal surface and consists of an oxide-hydroxide layer (Zn(OH)_2_ and ZnO) and a phosphonate layer represented by hardly soluble ZnDDP. This, along with intermolecular interaction (due to van der Waals forces), orientation, and ordering of alkyls in the surface layer, ensure its stability, and hydrophobic and anticorrosive properties. Despite this, the ultrathin phosphonate film does not provide long-term anticorrosive protection under severe conditions of 100% humidity and salt spray, as shown by the results of corrosion tests.

A low-intensity silicon doublet with a maximum of Si2*p* electrons at 102.0 eV is observed in the XPS spectrum after treatment of a “smooth” zinc sample (Type 1) with OTES. This indicates the chemisorption of OTES on the zinc surface due to the formation of Si-O-Zn metal–siloxane bonds. A peak appears in the region of high binding energies in the Auger ZnL_3_M_45_M_45_ spectrum as in the case of SDDP treatment. However, the Si-O-Zn maximum is at *E*_b_ = 499.2 eV, in contrast to the P-O-Zn peak (499.6 eV). The spectrum of O1*s* electrons can be decomposed into five peaks, three of which were encountered earlier (ZnO, Zn(OH)_2_ and H_2_O_ads_), and two can be attributed to the Si-O-Si bond and, probably, Si-O-Zn with maxima at 532.4 eV and 530.6 eV, respectively (Figure 7c). The peak attributed to Zn(OH)_2_ may also include a small fraction of the silanol component (Si-OH) of the formed thin silaxane layer, which is difficult to isolate due to the relatively small amount and close *E*_b_ values. Since the ratio of the integral intensities of peak [Si-O-Si]/[Si-O-Zn] equal to 2:1, and [O1*s*(Si-O-Zn)]/[Si2*p*] = 0.92:1, it can be assumed that one silanol group of the OTES molecule participates in the formation of the Si-O-Zn bond with zinc cations to form a (-Zn-O-Si-O-Zn-O-Si-) chain complex, and the other two form Si-O-Si bonds. The formation of a chain complex accompanied by a uniform distribution of electron density on silicon atoms can also be indicated by the small width of the Si2*p* peak. The calculated thicknesses of the surface layers were 2.8, 1.1, and 1.6 nm for siloxane film, Zn(OH)_2_ and ZnO, respectively.

The results obtained are consistent with the known mechanism of formation of polymer layers by TASs on the surface of various metals. Ethoxy groups of OTES are hydrolyzed in the presence of water to form reactive silanol groups (5), which interact with the hydroxylated zinc surface (6) in accordance with the scheme:

R-Si(OC_2_H_5_)_3_ + 3H_2_O → R-Si(OH)_3_ + 3C_2_H_5_OH (5)

R-Si(OH)_3_+ Zn(OH)_2_ → R-Si(OH)O_2_Zn + 2H_2_O (6)

where R is hydrocarbon radical. The resulting Si-O-Zn covalent metal–siloxane bonds contribute to the retention of the siloxane layer on the metal surface. It is assumed that the stability of this layer is largely determined by the formation of high-molecular crosslinked structures due to lateral interactions (between Si-OH groups with the formation of Si-O-Si siloxane bonds and Si-OH…HO-Si hydrogen bonds, as well as the van der Waals interaction between polar terminal groups) [3,26,61].

XPS studies of zinc samples sequentially treated in SDDP and OTES solutions showed the presence of interaction products of both OCIs on their surface. The position of the maximum of P2*p* electrons remained (133.6 eV), while the position of the Si2*p* peak shifted to the region of higher *E*_b_ values by 0.3 eV compared to the adsorption of only one OTES and amounted to 102.3 eV. There is also a change in the O1*s* spectrum (Figure 7d). The peak corresponding to the P=O bond shifted to the region of lower *E*_b_ values by 0.3 eV. This phenomenon can be caused by the formation of the Si-O-P bond, as a result of which the electron density is shifted from the silicon atom to a more electronegative oxygen atom. This along with lateral interactions between Si-OH groups and/or van der Waals interaction between polar alkyls leads to the formation of a high-molecular crosslinked layer. In addition, taking into account that the ratio of the integral intensities of peak [P2*p*]/[Si2*p*] equal to 1.43, the phosphonate component prevails in the surface layer. Thus, a thin phosphonate-siloxane film is formed, which, due to the combination of the barrier properties of the siloxane matrix with a strong chemical bond of phosphonic groups with the zinc surface, shows high protective properties compared to films obtained during passivation with individual OCIs. Despite the useful of the results obtained, for a clearer understanding of the phosphonate–siloxane film structure, it is necessary to carry out additional studies by IR or Raman spectroscopy, which we plan in our further studies.

**Figure 8:**
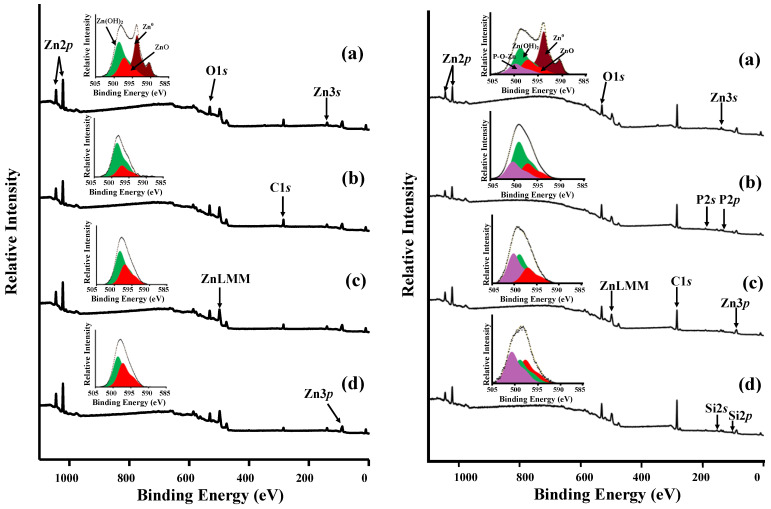
XPS survey spectra and Auger ZnL_3_M_45_M_45_ spectra of zinc samples with different surface types before (**left**) and after (**right**) layer-by-layer treatment in solutions of 2.5 mM SDDP and 10.0 mM OTES: (**a**)—Type 1, (**b**)—Type 2, (**c**)—Type 3, (**d**)—Type 4.

XPS analysis of zinc samples with different surface morphologies without treatment with OCIs showed a difference in the quantitative composition of the surface oxide-hydroxide layer (Table 5). In Auger ZnL_3_M_45_M_45_ spectra (Figure 8 left) obtained on the zinc surfaces textured by etching or laser (Types 2–4), there is no Zn^0^ metallic state, which indicates the formation of an oxide–hydroxide layer whose thickness exceeds the limit of the possible measurement depth in the XPS (>10 nm). After laser treatment, the fraction of Zn(OH)_2_ decreases in the surface layer, and fraction of ZnO increases. This is due to the dehydration of the natural oxide–hydroxide layer, as well as the oxidation of the metal under the exposure to laser radiation. On the contrary, the etching of zinc in the HCl solution leads to the formation of an oxide–hydroxide film on its surface with a larger amount Zn(OH)_2_ than ZnO.

According to XPS studies, the mechanism of SDDP and OTES on zinc surfaces with different morphologies is the same. Figure 8 (right) shows the spectra obtained on various surfaces with a layer-by-layer formed phosphonate–siloxane film. The position of the P2*p* and Si2*p* peaks does not depend on the type of the zinc surface. At the same time, an increase in their intensity is observed in the following order: Type 1 ˂ Type 2 ˂ Type 3 ˂ Type 4, which indicates an increase in the amount of phosphonate and siloxane components in the protective film with increasing in the roughness of the metal surface. Along with this, in the Auger ZnL_3_M_45_M_45_ spectrum of zinc samples with a phosphonate–siloxane layer formed on a previously etched surface (Type 2), the fraction of the Zn(OH)_2_ significantly exceeds the fraction of other components. The presence of a large amount of Zn(OH)_2_ in the protective layer negatively affects its protective properties compared to similar films on laser textured surfaces (Types 3 and 4), which is confirmed by the results of corrosion tests. This is also consistent with the regularities we obtained earlier [23]. Note that in the Auger ZnL_3_M_45_M_45_ spectra of zinc samples with a phosphonate–siloxane layer obtained on textured surfaces (Types 2–4), the position of the maximum corresponding to the P-O-Zn and Si-O-Zn bonds shifts to the region of a larger *E*_b_ (Δ*E*_b_ ≈ 0.6 eV) and reaches the highest value for the surface with fractal morphology (Type 4). In this case, the *E*_b_ values for ZnO and Zn(OH)_2_ do not change. Taking into account that the samples were analyzed after ultrasonic washing, this indicates a stronger adsorption of OCIs on the zinc surface textured by a laser at a low scanning speed, as well as the formation of a thicker and less defective protective film. The strong bond of the phosphonate–siloxane film with the zinc surface prevents its desorption in a corrosive media and, thus, ensures the preservation of protective properties for a long time.

## 4. Conclusions

1. Chemical etching or laser treatment at different scanning speeds allowed one obtain zinc surfaces that differ significantly in morphology, roughness, and composition of the surface oxide layer.
2. Differences in the wettability of the zinc surface with phosphonate–siloxane films depend in combination on its morphology and hydrophobicity of the OCIs used. All studied methods of surface texturing enhance the hydrophobic properties of thin phosphonate–siloxane films. However, superhydrophobic films are obtained either by layer-by-layer passivation of SDDP with a more hydrophobic silane (OTES) or on the surface with fractal morphology obtained by laser treatment at the low scanning speed.
3. Hydrophobization or even superhydrophobization of zinc with phosphonate–siloxane films does not always ensure its high corrosion resistance in corrosive atmospheres. Phosphonate–siloxane films obtained on textured zinc surfaces by etching and laser at the high scanning speed are characterized by similar wetting characteristics, despite a significant difference in protective properties.
4. Preliminary laser treatment of the zinc surface is more effective than chemical etching to enhance the anticorrosive properties of the resulting thin films.
5. According to XPS studies, the mechanism of adsorption of SDDP and OTES on zinc surfaces with different morphology is the same. The thin phosphonate–siloxane film obtained by layer-by-layer technique consists of layers of chemisorbed zinc phosphonate, siloxane, ZnO and Zn(OH)_2_. The fraction of phosphonate and siloxane components in the protective film increases with roughness of the zinc surface. The thickest and least defective phosphonate–siloxane film, strongly bonded to the surface, is formed on the surface with fractal morphology enriched with ZnO. The combination of the barrier properties of a polysiloxane matrix with a strong chemical bond of phosphonic groups with the zinc surface prevents desorption of OCIs and provides high protective properties of this film during long-term corrosion tests in corrosive atmospheres of high humidity and salt spray.

## Figures and Tables

**Table 1:** Roughness parameters of zinc samples with different types of surface.

Surface Types		*R*_z_, µm	*R*_a_, µm	*S*, µm	Roughness Class
Type 1		1.70	0.09	4.26	3
Type 2	without heat treatment	9.66	0.30	23.60	6
	with heat treatment	7.67	0.21	21.32	6
Type 3	without heat treatment	16.40	1.58	7.98	7
	with heat treatment	15.85	1.38	5.99	7
Type 4	without heat treatment	33.45	5.80	9.10	9
	with heat treatment	32.32	4.43	7.69	9

**Table 2:** The elemental composition of the zinc surface with different texturing methods, determined by EDX analysis.

Surface Types		Element, wt.%	
		O	Zn
Type 1		1.09	98.91
Type 2		1.06	98.94
Type 3	without heat treatment	2.06	97.94
	with heat treatment	1.08	98.92
Type 4	without heat treatment	11.14	88.86
	with heat treatment	10.49	89.51

**Table 3:** Θ_c_ values on “smooth” (Type 1) and textured (Types 2–4) zinc surfaces without and with treatment in SDDP and TASs solutions.

Composition of the Inhibiting Solution, mM	Θ_c_, °			
	Surface Types			
	Type 1	Type 2	Type 3	Type 4
without treatment with OCI	72 ± 3	125 ± 1	120 ± 3	155 ± 1
2.5 SDDP	95 ± 2	129 ± 2	134 ± 2	138 ± 3
10.0 VTMS	91 ± 3	134 ± 2	135 ± 3	140 ± 3
10.0 OTES	89 ± 2	149 ± 1	148 ± 2	159 ± 2
2.5 SDDP///10.0 VTMS	102 ± 3	145 ± 2	142 ± 2	160 ± 2
2.5 SDDP///10.0 OTES	124 ± 2	155 ± 2	159 ± 1	165 ± 3

**Table 4:** γ and *Z* values calculated based on the results of corrosion tests in aggressive atmospheres of zinc samples with different surface morphology, treated in SDDP and TASs solutions.

Composition of the Inhibiting Solution, mM	γ///*Z*, %			
	Surface Types			
	Type 1	Type 2	Type 3	Type 4
In a heat and moisture chamber				
Without treatment with OCI	–	–	–	–
2.5 SDDP	9.2///89.1	8.9///88.8	4.2///76.0	5.8///82.9
10.0 VTMS	8.2///87.8	8.5///88.2	11.8///91.5	34.5///97.1
10.0 OTES	10.4///90.4	11.9///91.6	14.3///93.0	49.4///98.0
2.5 SDDP///10.0 VTMS	27.8///96.4	27.5///96.4	39.4///97.5	57.6///98.3
2.5 SDDP///10.0 OTES	29.3///96.6	28.5///96.5	44.5///97.8	64.4///98.4
In a salt spray chamber				
Without treatment with OCI	–	–	–	–
2.5 SDDP	4.5///77.8	5.0///80.0	5.5///81.8	8.0///87.5
10.0 VTMS	15.0///93.3	15.5///93.5	16.5///93.9	44.5///97.8
10.0 OTES	17.5///94.3	20.5///95.1	21.0///95.2	65.5///98.5
2.5 SDDP///10.0 VTMS	34.5///97.1	38.0///97.4	56.0///98.2	80.0///98.8
2.5 SDDP///10.0 OTES	48.5///97.9	49.0///98.0	65.0///98.5	105.5///99.1

**Table 5:** Composition of the surface oxide-hydroxide layer on “smooth”, chemically etched and laser textured zinc samples.

Surface Types	Element Concentrations (%)		
	O1*s*		Zn2*p*
	ZnO	Zn(OH)_2_	
Type 1	24.00	39.50	36.50
Type 2	16.50	42.70	40.80
Type 3	24.50	37.80	37.70
Type 4	33.30	24.00	42.70

## Data Availability

Not applicable.

## Appendix A

**Table A1:** List of most frequent abbreviations and symbols.

**Symbols**			
log*P*	logarithm of the distribution coefficient of the substance in the water–octanol system (for neutral molecules)	*τ_cor_*	time to the appearance of the first corrosion damage
*V*	laser scanning speed	$\tau_{\mathrm{cor}}^{0}$	time of appearance of the first corrosion damage on the zinc surface without passivation
γ	coefficient of corrosion retardation	$\tau_{\mathrm{cor}}^{\mathrm{pas}}$	time of appearance of the first corrosion damage on the zinc surface after passivation with OCIs
Z	degree of protection	Θ_c_	water contact angle
*R*_z_	height of profile irregularities at ten points	*E*_cor_	corrosion potential
*R*_a_	the mean arithmetic deviation of the profile	$E_{\mathrm{pit}}^{\mathrm{OCI}}$	pitting potential on samples without treatment with OCIs
*S*	the average step of the local projections of the profile within the base length	$E_{\mathrm{pit}}^{\mathrm{OCI}}$	pitting potential on samples with preliminary passivation with OCIs
**Abbreviations**			
OCI	organic corrosion inhibitor	SAM	self-assembled monolayer
TAS	trialkoxysilane	C_n_PA	alkylphosphonic acid
VTMS	vinyltrimethoxysilane	SDDP	sodium dodecylphosphonate
OTES	*n*-octyltriethoxysilane		
